# Beneficial Effects of Metformin on the Central Nervous System, with a Focus on Epilepsy and Lafora Disease

**DOI:** 10.3390/ijms22105351

**Published:** 2021-05-19

**Authors:** Pascual Sanz, José Maria Serratosa, Marina P. Sánchez

**Affiliations:** 1 Instituto de Biomedicina de Valencia (CSIC), Centro de Investigación Biomédica en Red de Enfermedades Raras (CIBERER), Jaime Roig 11, 46010 Valencia, Spain; 2 IIS Fundación Jiménez Díaz, Centro de Investigación Biomédica en Red de Enfermedades Raras (CIBERER), 28040 Madrid, Spain; joseserratosa@icloud.com

**Keywords:** AMPK, epilepsy, GPD2, Lafora disease, mechanism of action, metformin, neurological disorders

## Abstract

Metformin is a drug in the family of biguanide compounds that is widely used in the treatment of type 2 diabetes (T2D). Interestingly, the therapeutic potential of metformin expands its prescribed use as an anti-diabetic drug. In this sense, it has been described that metformin administration has beneficial effects on different neurological conditions. In this work, we review the beneficial effects of this drug as a neuroprotective agent in different neurological diseases, with a special focus on epileptic disorders and Lafora disease, a particular type of progressive myoclonus epilepsy. In addition, we review the different proposed mechanisms of action of metformin to understand its function at the neurological level.

## 1. Introduction

Metformin (*N*,*N*-Dimethylimidodicarbonimidic diamide) belongs to the family of biguanide compounds that have glucose-lowering effects. Among the biguanide family of compounds, metformin was initially of little clinical interest, due to its low potency requiring high doses of the compound to be effective. However, metformin showed a higher safety profile than its counterparts, such as phenformin or buformin, which were discarded for clinical use because they produced lactic acidosis (reviewed in [1]). Metformin is currently the most commonly prescribed drug for type 2 diabetes (T2D), and is taken by an estimated 150 million people worldwide. It has the advantage over other non-insulin-based diabetes therapies of reducing blood glucose levels without inducing hypoglycemia. Due to its superior safety profile, it has become the first-line treatment for T2D, and is now featured on the World Health Organization’s essential medicines list [1,2,3]. However, although metformin is usually well tolerated, it does have some side effects. In some patients, it can produce lactic acidosis, gastrointestinal discomfort, and vitamin B12 deficiency. For this reason, metformin should be administered initially at low doses that are increased if side effects do not appear [4].

The regular dose of metformin used in diabetic patients is 1–2 g/day, leading to a plasma metformin concentration of 50–100 μM. Numerous reports on the possible metformin mechanisms of action have been published, and some contradictory results have been shown due to differences in the cellular system used in the analyses, or because different doses of metformin were used (reviewed in [1]). Thus, different mechanisms of action have been described depending on the dose used in rats and mice: (1) supra-pharmacological metformin doses (>250 mg/kg/day) achieved >1 mM plasma metformin concentration; and (2) regular pharmacological treatment (50–100 mg/kg/day) achieved 50–100 μM metformin concentration, which corresponds to the regular human dose mentioned above [1,2,3]. Thus, depending on the dose, the mechanism of action of metformin can be explained by it affecting different signaling pathways (see below).

**Figure 1:**
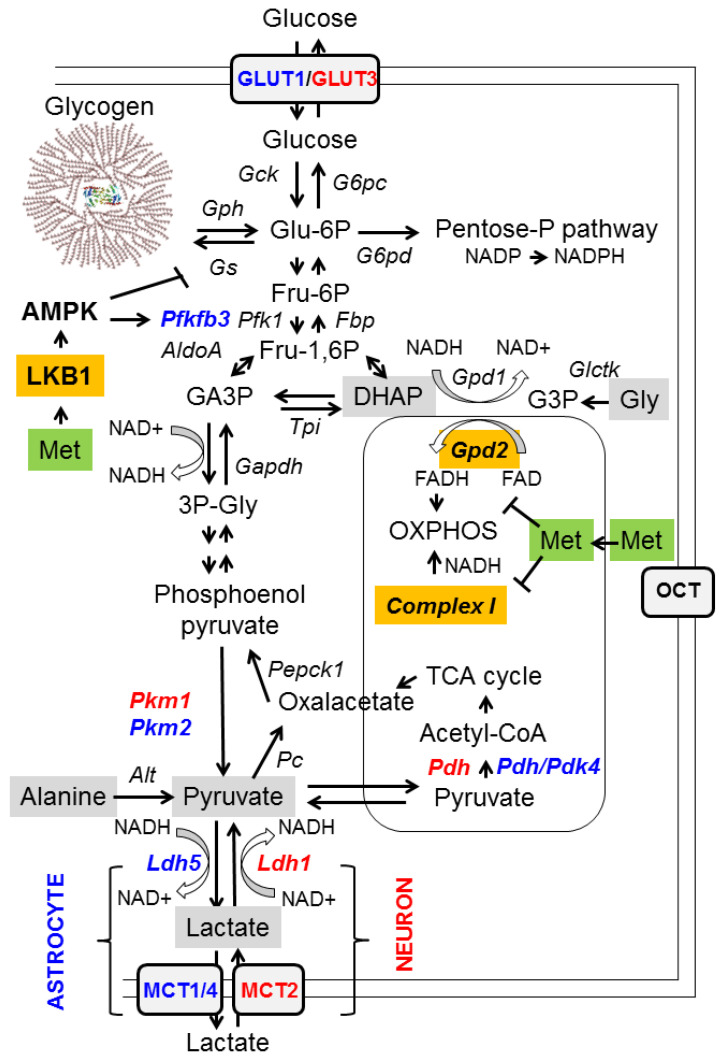
Metabolic pathways affected by metformin. A diagram of the main pathways related to glycolysis and gluconeogenesis is depicted. Gluconeogenic substrates are highlighted in grey, and enzymes directly affected by metformin are highlighted in orange. Specific enzyme isoforms present in neurons are in red, and those present in astrocytes are in blue. See text for details. AldoA: aldolase; Alt: Alanine aminotransferase; AMPK: AMP-activated protein kinase; NADH: ubiquinone oxidoreductase; Fbp: fructose bisphosphatase; Gapdh: glyceraldehyde-3P dehydrogenase; G6pc: glucose 6-phosphatase; G6pd: glucose-6P dehydrogenase; Gck: glucokinase; Glctk: glycerate kinase; GLUT: glucose transporter; Gpd: glycerol-3P dehydrogenase; Gph: glycogen phosphorylase; Gs: glycogen synthase; Ldh: lactate dehydrogenase; LKB1: liver protein kinase; MCT: monocarboxylic transporter; OCT: organic cation transporter; Pc: pyruvate carboxylase; Pdh: pyruvate dehydrogenase; Pdk: pyruvate dehydrogenase kinase; Pepck1: phosphoenolpyruvate carboxykinase; Pfk: phosphofructokinase; Pfkfb3: phosphofructokinase 2,6-bisphosphatase; Pkm: pyruvate kinase; TCA cycle: tricarboxylic acid cycle; Tpi: triosephosphate isomerase.

According to PubChem [PubChem. Available online: http://pubchem.ncbi.nlm.nih.gov/compound/metformin (accessed on 12 May 2021)], the pKa of metformin is 12.4. This value indicates that metformin exists in a monoprotonated form at neutral pH (6–8), and that only when it reaches a pH < 3.1 would it exist as biprotonated species. Due to its unusually hydrophilic nature, metformin cannot passively diffuse through cell membranes, and must rely on members of organic cation transporters (OCTs) [1,2,3]. Therefore, only those tissues that express members of the OCT family (e.g., liver, kidney, and small intestine) will be targets for the action of metformin [5,6,7]. Metformin can also act on neuronal cells as they express two members of the OCT family, namely OCT1 and plasma membrane monoamine transporter (PMAT) [8]. Once metformin enters cells, it accumulates within the mitochondria [1] (Figure 1).

The therapeutic potential of metformin treatment expands its prescribed use as an anti-diabetic drug. Thus, metformin has been shown to be effective in the treatment of multiple diseases, including polycystic ovary syndrome [9], cardiovascular disease [10,11,12], and cancer [12,13,14]. In addition, it delays the aging process [12,15,16] and alleviates inflammation associated with it [17], and also modulates the microbiota to promote health [3,18]. Furthermore, metformin may have additional beneficial effects yet to be discovered. The beneficial effects of metformin also extend to neurodegenerative diseases; metformin has been reported to alleviate the pathophysiology of Alzheimer’s, Parkinson’s, and Huntington’s diseases, as well as multiple sclerosis, among others [12,19]. In this work, we will review the different proposed mechanisms of action of metformin and the beneficial effect that this drug has as a neuroprotective agent in different neurological diseases, with special attention to epileptic disorders and Lafora disease, a form of progressive myoclonus epilepsy (see below).

## 2. Proposed Mechanism of Action of Metformin to Lower Glucose Levels

### 2.1. Inhibition of Mitochondrial Glycerol-3-Phosphate Dehydrogenase (GPD2)

It is now widely accepted that the anti-hyperglycemic effect of metformin is mainly due to the suppression of hepatic glucose production (HGP) (reviewed in [1,2,3]). Hepatic glucose production is the result of the balance between glucose forming pathways (gluconeogenesis and glycogenolysis) and glucose consuming pathways (glycogen synthesis, glycolysis, and the pentose phosphate pathway) (Figure 1). Among them, hepatic gluconeogenesis contributes more than 50% of HGP and is considered the main pathway regulated by metformin. Metformin negatively regulates gluconeogenesis at different levels:

(a) At the transcriptional level: metformin prevents cAMP responsive binding (CREB)-mediated transcription of the gluconeogenic glucose 6-phosphatase (*G6PC*) and phosphoenolpyruvate carboxykinase 1 (*PEPCK1*) genes. This is an indirect effect due to the inhibition of mitochondrial complex I by metformin (see below), leading to an increase in AMP levels, which inhibits adenylate cyclase and thus leads to a decrease in levels of cAMP, a mediator of CREB-dependent transcription. In addition, metformin has been proposed to activate AMP-activated protein kinase (AMPK), which has a negative effect on the transcriptional regulation of gluconeogenesis genes, among others [4] (see below);
(b) Reducing the availability of gluconeogenic substrates: hepatic gluconeogenesis depends on the availability of appropriate substrates, such as glycerol, lactate, pyruvate, alanine, and dihydroxyacetone phosphate (DHAP), in order to convert them to glucose (Figure 1, grey boxes). Glycerol and DHAP are mutually interconnected, since glycerol is converted to glycerol 3-P (G3P) by glycerate kinase (Glctk) and then G3P is converted to DHAP by mitochondrial glycerol-3-phosphate dehydrogenase (GPD2). Metformin has been shown to inhibit mitochondrial GPD2 at regular concentrations (50–100 μM) (reviewed in [1,2,3]; Figure 1, orange box). Therefore, after treatment with metformin, the levels of DHAP are reduced, and this leads to a decrease in the flux of gluconeogenesis. As a consequence of this inhibition, G3P and glycerol accumulate in hepatocytes (Figure 1).

The function of GPD2 is coupled to one of the major NADH/NAD+ shuttles, the alpha-glycerophosphate shuttle, which consumes NADH and transforms DHAP to glycerol-3P through the action of cytosolic glycerol-3P dehydrogenase 1 (GPD1). Inhibition of GPD2 by metformin alters the cytosolic redox balance and leads to a higher NADH/NAD+ ratio, due to low levels of DHAP. This high NADH/NAD+ ratio also prevents the conversion of lactate into pyruvate by lactate dehydrogenase (LDH). Therefore, inhibition of GPD2 by metformin decreases the levels of two of the main gluconeogenic substrates, DHAP and pyruvate, and leads to the accumulation of glycerol and lactate (Figure 1). This accumulation of lactate is probably the cause of the appearance of lactic acidosis in some patients treated with metformin [1,2,3].

However, metformin does not affect the gluconeogenic use of pyruvate and alanine as substrates, since their entry into the gluconeogenesis pathway does not involve a redox-dependent mechanism (Figure 1). This would explain why hypoglycemia is rarely observed in patients treated with metformin or in healthy individuals, since part of the gluconeogenesis pathway is still active [1].

### 2.2. Inhibition of Mitochondrial Complex I of the Respiratory Chain and Activation of AMP-Activated Protein Kinase (AMPK)

An additional mechanism to explain the hypoglycemic effect of metformin on hepatocytes is its inhibitory action on mitochondrial complex I (NADH/ubiquinone oxidoreductase) [20]. Mitochondrial complex I of the respiratory chain is the site of the contribution of NADH to the proton gradient of OXPHOS (oxidative phosphorylation) (Figure 1, orange box). Inhibition of complex I by metformin reduces the mitochondria’s ability to consume NADH and the production of ATP. High levels of NADH and low levels of ATP have a crucial negative impact on the gluconeogenesis pathway, as this process requires a large amount of energy and depends on a correct NADH/NAD + balance. Furthermore, as ATP production is reduced, the AMP/ATP ratio increases, and this leads to the activation of AMP-activated protein kinase (AMPK), a master regulator of energy homeostasis [2,4,21]. AMPK activation leads to the activation of catabolic pathways (e.g., glycolysis through the activation of Pfkfb3, an enzyme involved in the formation of 2,6-fructose bisphosphate, an allosteric activator of phosphofructokinase 1 (Pfk1)) and the inhibition of anabolic pathways (e.g., glycogen synthesis by inhibiting glycogen synthase; Gs), to restore energy balance (Figure 1) [4,21]. AMPK exerts this function both at the transcriptional level, regulating the activity of different transcriptional factors by phosphorylation (e.g., the downregulation of CREB, carbohydrate-responsive element binding protein (ChREBP), and sterol regulatory element binding protein (SREBP-1), which are involved in the expression of genes related to gluconeogenesis, the carbohydrate metabolism, and sterol biosynthesis, respectively; on the other hand, AMPK upregulates peroxisome proliferator-activated receptor γ co-activator 1 alpha (PGC1alpha), involved in mitochondrial biogenesis, and activates pro-health span molecules, such as the forkhead box O3 (FOXO3) transcription factor and sirtuin 1 (SIRT1) deacetylase, which in turn induce the expression of protective molecules [2,4,21,22]). AMPK also operates at the level of key metabolic enzyme activity (e.g., inhibition of acetyl-Co carboxylase (Acc1/2), an enzyme involved in the synthesis of malonyl-CoA, an intermediate in fatty acid synthesis, and an inhibitor of fatty acid oxidation; therefore, AMPK activation inhibits the synthesis of fatty acids and promotes their degradation) [21]. Therefore, the increase in the AMP/ATP ratio caused by metformin activates AMPK indirectly, but this effect is only obtained when metformin is administered at supra-pharmacological concentrations (>1 mM) [1]. Recent results support the indirect effect of metformin on the activation of AMPK, as they show that metformin does not affect AMPK directly, but acts on the upstream liver kinase (LKB1), which participates in the phosphorylation and activation of the catalytic alpha subunit of AMPK [23] (Figure 1, orange box).

### 2.3. Effects of Metformin on Glucose Metabolism in the Brain

The effect of metformin on glucose metabolism occurs not only in hepatocytes, but also in other tissues, such as the brain [4]. Neurons and astrocytes are recognized to have different glucose metabolic profiles. The metabolism of neurons relies more on mitochondrial oxidative phosphorylation (OXPHOS) [24,25], due to the singularities of different glycolytic enzymes present in these cells. Thus, neurons have a pyruvate kinase isoform (Pkm1) that is completely active, and the resulting pyruvate is rapidly transformed into acetyl-CoA by active mitochondrial pyruvate dehydrogenase (Pdh) (Figure 1, enzymes highlighted in red) [24,25]. On the other hand, neurons express a lactate dehydrogenase isoform (Ldh1) that works better by transforming lactate into pyruvate, so the amount of lactate produced by them is very low. Furthermore, they express the monocarboxylate transporter 2 (MCT2) which is fully active in the uptake of lactate from the surrounding medium [24,25] (Figure 1, enzymes highlighted in red). In conclusion, neurons are prepared to capture lactate and convert it into pyruvate, which will enter the mitochondria to obtain energy. In contrast, astrocytes have a glucose metabolism that is more glycolytic [24,25]. They have reduced OXPHOS due to the low activity of their mitochondrial pyruvate dehydrogenase (Pdh), since it is inactivated by an active pyruvate dehydrogenase kinase 4 (Pdk4) (Figure 1, enzymes highlighted in blue). In addition, astrocytes express an isoform of lactate dehydrogenase (Ldh5) that is fully active in reducing pyruvate to lactate, which is exported outside the cell by monocarboxylate transporters MCT1/4. Hence, the glucose metabolism of astrocytes is designed to produce lactate and export it to the surrounding media. In addition, astrocytes can enhance glycolytic flux, because they have an active phosphofructokinase 2,6-bisphosphatase (Pfkfb3) that synthesizes 2,6-fructose bisphosphate, a potent allosteric activator of phosphofructokinase 1 (Pfk1) (Figure 1, enzymes highlighted in blue), and this enzyme can be fully activated by AMPK [24,25] (see above).

Bearing all this information in mind, we hypothesize that at the central nervous system (CNS), metformin would produce a reduction in gluconeogenesis in both neurons and astrocytes by inhibiting mitochondrial Gpd2 and activating AMPK, which would cause a decrease in the levels of DHAP and pyruvate, as in the case of hepatocytes (see above); in addition, this would cause an increase in the glycolytic flux (use of Glu-6P). This would in turn lead to increased lactate production in astrocytes and increased OXPHOS in neurons. Glycolysis would also increase in astrocytes, due to the metformin-mediated activation of AMPK that would activate Pfkfb3, leading to an enhancement of Pfk1 activity (Figure 1). To cope with a higher demand for Glu-6P, glucose uptake and glycogen degradation would be accelerated. As metformin-mediated activation of AMPK would also inactivate glycogen synthase, we expect glycogen levels to decrease after metformin treatment. This could explain the prevention in polyglucosan synthesis in mouse models of Lafora disease after treatment with metformin [26] (see below).

### 2.4. Metformin Ameliorates Oxidative Stress

Substantial evidence shows that metformin exerts antioxidant effects. Some of these can be attributed to the inhibition of mitochondrial complex I, which reduces reactive oxygen species (ROS) production by the OXPHOS respiratory chain [4]. In addition, metformin has other functions related to the activation of the AMPK pathway: (i) reduction of ROS levels by upregulating the expression of antioxidant enzymes, such as thioredoxin, through the AMPK–FOXO3 pathway; (ii) modulation of the expression of sirtuin 3 (SIRT3) deacetylase, whose activity promotes antioxidant effects in the cell; (iii) downregulation of NADPH oxidase, one of the main producers of cellular ROS; and (iv) enhancement of mitochondrial biogenesis by enhancing the function of PGC1alpha transcription factor [27].

### 2.5. Metformin and Neuroinflammation

Following brain injury, neuroinflammation is initially neuroprotective, but when it becomes chronic or excessive, it eventually causes damage [28]. It is now accepted that sustained brain inflammation promotes neuronal hyperexcitability and seizures, and that dysregulation in the immunoinflammatory function of the glia is a common factor that predisposes or contributes to the generation of seizures. At the same time, acute seizures upregulate the production of pro-inflammatory cytokines in microglia and astrocytes, triggering a cascade of inflammatory mediators. Thus, epileptic seizures and inflammatory mediators form a positive feedback loop, reinforcing each other [28]. For this reason, it has recently been proposed that the treatment of inflammation with specific anti-inflammatory drugs may be beneficial in the treatment of refractory epilepsies [28]. However, since the use of general anti-inflammatory drugs is not recommended due to their detrimental performance in long-term treatments [29], only specific anti-inflammatory compounds, whose selection has been made after a thorough understanding of the main related inflammatory pathways, should be used with each particular type of epilepsy.

Activation of nuclear factor kappa-light-chain-enhancer of activated B cells (NF-kB) is a hallmark of neuroinflammation, and is present in most neurological diseases. The Toll-like receptor 4 (TLR4) signaling pathway induces NF-kB activation through myeloid differentiation primary response 88 (MyD88) and tumor necrosis receptor-associated factor 6 (TRAF6), leading to the expression of pro-inflammatory mediators: cytokines, chemokines, cyclooxygenase 2 (COX2), and inducible nitric oxide synthase (iNOS) [30]. It has been described that the activation of AMPK by metformin reduces general inflammatory conditions since it inhibits the signaling of NF-kB, as well as the expression of pro-inflammatory cytokines (interleukin 1-beta (IL-1beta), interleukin 6 (IL-6), tumor necrosis factor alpha (TNFalpha), C–C motif chemokine ligand 2 (CCL2), etc.) in different cell types [31,32,33], suggesting that AMPK activation could protect against neuroinflammation [19]. Similarly, activation of AMPK by berberine reduces activated microglia; neutrophil infiltration; and IL-1beta, IL-6, CCL2, and CXCL2 production, which occur after traumatic brain injury [34]. In both cases, AMPK prevented the activation of the TLR4/NF-kB signaling pathway [31,34,35]. AMPK also inhibits lipopolysaccharide (LPS)-induced expression of proinflammatory cytokines (TNF-alpha, IL-1beta, and IL-6) by attenuating LPS-induced, TLR4-mediated NF-kB activation [36,37,38]. Similarly, AMPK prevented the advanced glycation end-product (AGE)-mediated signaling pathway, which ends with an increase in NF-kB expression and reduced iNOS and COX2 levels in AGE-treated human neural stem cells (hNSCs) [39]. The anti-inflammatory action of AMPK was also associated with the inhibition of LPS-induced activation of the phosphatidyl inositol 3 kinase (PI3-kinase)/RAC-alpha serine/threonine-protein kinase (Akt) pathway [37]. Downregulation of NF-kB levels inhibits the activation of the nucleotide-binding oligomerization domain and leucine-rich repeat and pyrin domain 3 (NLRP3) inflammasomes, while decreasing the activation of caspase1 and reducing the production of IL-1beta [38]. These mechanisms are particularly important in microglia, where AMPK inhibits the release of pro-inflammatory markers, decreasing neuroinflammation [40], and in astrocytes, where AMPK inhibits elevated ER stress and hyperglycemia-induced inflammation [41].

Since neuroinflammation is a recognized event associated with neurological disorders, such as Alzheimer’s, Parkinson’s, and Huntington’s diseases, and epilepsy, metformin could have a positive effect on these diseases (see below).

## 3. Metformin as a Neuroprotective Agent in Different Neurological Disorders

There is growing interest in the potential use of metformin in diseases of the central nervous system (CNS). Although most neurological disorders are different in nature, they share basic pathological mechanisms that are altered in the corresponding disease. Examples of these are the AMPK and mechanistic target of rapamycin (mTOR) kinase pathways. As indicated above, AMPK is a master regulator of energy homeostasis. It is activated in conditions of energy deprivation, and by activating catabolic pathways and inhibiting anabolic pathways it restores energy balance. In contrast, the mTOR pathway is activated under high-energy conditions, and operates by activating anabolic pathways and inhibiting catabolic pathways. Both the AMPK and mTOR pathways are interconnected, and activation of the AMPK pathway results in inhibition of the mTOR system, either by directly inactivating components of the mTOR complex (e.g., raptor, tuberous sclerosis complex 2 (TSC2)) or by reversing the effect of mTOR on common substrates (e.g., ULK1 in the autophagy process) [42]. In particular, metformin activates AMPK signaling, and also inhibits mTOR signaling via AMPK-dependent as well as AMPK-independent pathways [1,2,3]. In the following, we will briefly review the action of metformin as a neuroprotector agent in various neurological disorders.

### 3.1. Alzheimer’s Disease

Alzheimer’s disease (AD) is the most prevalent neurodegenerative disorder. It is characterized by progressive memory loss and impaired cognitive function. Clinical studies indicate an ameliorative effect of metformin on cognitive decline and Alzheimer’s disease [43]. In fact, patients with T2D receiving metformin had a lower risk of cognitive impairment and a lower risk of developing Alzheimer’s disease than other patients with T2D receiving alternative treatments [44]. AMPK activation has also been reported to have neuroprotective effects in different mouse models of Alzheimer’s disease [45,46].

### 3.2. Parkinson’s Disease

Parkinson’s disease is the second most prevalent neurodegenerative disorder. Disease progression has been considered a consequence of mitochondrial dysfunction, with elevated levels of reactive oxygen species (ROS) and increased oxidative stress leading to the death of dopaminergic neurons [47]. Treatment with metformin reduces oxidative stress and improves the expression of antioxidant enzymes, such as superoxide dismutase and catalase [48]. This effect of metformin was achieved both by activating the AMPK pathway as well as through AMPK-independent mechanisms, such as activation of the brain-derived neurotrophic factor (BDNF) signaling pathway [49]. In animal models of Parkinson’s disease, metformin inhibits alpha-synuclein aggregation, prevents mitochondrial dysfunction, attenuates oxidative stress, enhances autophagy through AMPK activation, and prevents neurodegeneration and neuroinflammation (reviewed in [50]).

### 3.3. Huntington’s Disease

Huntington’s disease is an autosomal, dominant inherited disorder related to the presence of a defective huntingtin gene (*Htt*) [51]. Mutant huntingtin protein containing long poly-Q tracks overloads the ubiquitin proteasomal degradation system and forms aggregates with itself and with other proteins, leading to a depletion of critical molecules involved in neuronal homeostasis and resulting in neuronal degeneration [51]. Treatment with metformin has been reported to reduce the number of huntingtin aggregates, probably due to activation of autophagy, which results from activation of the AMPK pathway [43].

The reduction in the number of huntingtin aggregates is correlated with an improvement in cognitive and behavioral function in mouse models of Huntington’s disease [52].

### 3.4. Multiple Sclerosis

Multiple sclerosis is a chronic autoimmune disease that causes demyelination and destruction of neuronal cells at the CNS [53]. Current therapeutic approaches are based on the regulation of autoimmune attacks and the preservation of oligodendrocyte function. Metformin alleviates oxidative stress and restores mitochondrial function in patients with multiple sclerosis [54,55]. Furthermore, in an AMPK-dependent manner, metformin was able to enhance the expression of genes involved in the protection of oligodendrocytes and the restoration of central nervous system functions in an experimental model of autoimmune encephalomyelitis [56].

### 3.5. Epilepsy

Epilepsy is a neurological disorder characterized by a predisposition to generate epileptic seizures and the associated cognitive, psychological, and social consequences of this condition [28,57]. Epilepsy affects 1% of the total world population (around 65 million people worldwide) and is caused by acquired injuries in the brain (for example, after a stroke or traumatic brain injury), infectious diseases, autoimmune diseases, and genetic mutations. To date, more than 500 genes are associated with epilepsy [57]. The first-line treatment for epilepsy is antiseizure drugs (ASDs). The development of ASDs was based on the neuron-centric hypothesis that an imbalance of excitatory and inhibitory currents was largely responsible for epileptic seizures [58]. However, despite the availability of many ASDs, approximately one-third of patients fail to control seizures or soon become resistant to the effects of the ASDs [57,58]. Consequently, there is a critical need to develop innovative antiepileptic treatment strategies to improve progression and limit the detrimental consequences of the disease.

Since some forms of epilepsy are related to the upregulation of the mTOR pathway, different therapeutic strategies have been designed to inhibit this pathway [59]. The inhibition of mTOR signaling promotes a reduction in the generation of pro-inflammatory cytokines and chemokines (IL-1beta, TNFalpha, CCL2, iNOS, etc.) by microglia, which results in improvements in the motor deficit in the middle cerebral artery occlusion (MCAO) model of cerebral stroke [60]. As indicated above, AMPK is a master regulator of energy homeostasis: it is activated under conditions of energy deprivation, and by activating catabolic and inhibiting anabolic pathways it restores energy balance. In contrast, the mTOR pathway is activated under high-energy conditions, and operates by activating anabolic pathways and inhibiting catabolic pathways. Activation of these pathways occurs at the CNS, as with other peripheral tissues [4]. Interestingly, the AMPK and mTOR pathways are interconnected, and the activation of the AMPK pathway results in the inhibition of the mTOR system (see above). In particular, activation of AMPK by metformin inhibits mTOR signaling and has resulted in improved seizure control in models of mTOR overactivation [1,3,61,62]. 

These results were confirmed by a recent report indicating that AMPK activation by metformin improved lithium- and pilocarpine-induced status epilepticus in rats by inhibiting the mTOR pathway [63]. The beneficial effect of AMPK activation by metformin and calorie restriction on the main symptoms of an electric-ignition model of epilepsy, especially those related to generalized seizures, has also been observed [64].

Using an epileptic diabetic rat model, the activation of AMPK by metformin was reported to improve the inflammatory status and histopathological alterations present in this model [65]. Metformin normalized the levels of glutamate and gamma-aminobutyric acid (GABA), and reduced the levels of IL-1beta, TNF-alpha, NF-kB, and caspase 3. These effects were proposed to be a consequence of AMPK activation and mTOR inhibition [65].

AMPK activation also has beneficial effects on other models of epilepsy. For example, metformin has a beneficial effect against seizures and epilepsy in mouse models of induced epilepsy with pentylenetetrazol (PTZ), a pro-convulsive agent, due to its antioxidant and anti-apoptotic actions and upregulation of heat shock protein 70 (Hsp70) [62,66,67]. The anti-apoptotic effect was related to a downregulation of the RNA-like endoplasmic reticulum kinase (PERK) pro-apoptotic protein kinase/eukaryotic initiation factor 2 alpha (eIF2alpha)/activating transcription factor 4 (ATF4)/C/EBP homologous protein (CHOP) pathway [39]. Recently, it has been described that metformin reduces CHOP expression and apoptosis induced by status epilepticus in rats [68]. The beneficial effects of metformin on this model could also be due to an improvement in autophagy [69]. Additional reports indicate that AMPK activation by metformin decreases seizure susceptibility, facilitates seizure termination, and reduces the number and duration of seizures in a PTZ-induced epilepsy model [70].

AMPK activation by metformin also exhibits an anti-inflammatory effect in kainate-induced status epilepticus by inhibiting IL-1beta production and reducing the expression of glial fibrillary acidic protein (GFAP) and S100beta markers of astrogliosis, as well as by increasing secretion of the anti-inflammatory cytokine IL-10 [71]. In this model, metformin exerted a neuroprotective role against the kainate-induced epileptogenic process by preventing neuronal cell death, aberrant neurogenesis, and mossy fiber sprouting [72].

An additional relationship between AMPK and epilepsy is seen through the regulatory action on glucose transporter GLUT1. GLUT1 is the main glucose transporter in endothelial and astrocytic cells. GLUT1 deficiencies disrupt glucose transport in the brain, and this leads to seizures in patients with GLUT1 deficiency syndrome (OMIM 606777) [73]. AMPK activation has recently been shown to regulate the translocation of GLUT1 to the plasma membrane from internal stores by destabilizing the thioredoxin-interacting protein (TXNIP). This enhances astrocytic glucose uptake and glycolysis and enables proper regulation of the astrocyte–neuron lactate shuttle (ANLS), which preserves neuronal metabolic functionality [74]. This could explain the beneficial effects of AMPK activation by metformin on PTZ-induced seizures in mice on a high-fat diet, since after treatment with metformin, a normalization in the expression levels of GLUT1 and GLUT3 was observed [75].

In conclusion, AMPK activation was able to attenuate the generation of seizures by delaying the onset of epilepsy, reducing neuronal loss in the hippocampus, and preventing cognitive impairments in both acute and chronic epilepsy models. In the case of metformin, its antiepileptic effects could be attributed to an amelioration of oxidative brain damage, activation of the AMPK pathway, inhibition of the mTOR signaling, downregulation of brain-derived neurotrophic factor (BDNF), and neurotrophic receptor tyrosine kinase 2 (TrkB) levels or improvement of proteostasis [76,77].

Therefore, AMPK can be considered as a new anti-inflammatory and antiepileptic signaling pathway. Its activation leads to a decrease in the mTOR and TLR4/NF-kB signaling pathways, thus representing a promising therapeutic target for immunoinflammatory disorders like epilepsy [78]. In fact, in a recent screening of repurposing drugs with anticonvulsive properties, AMPK activators, such as metformin, appeared as promising candidates with therapeutic potential as anti-epileptic drugs [79].

## 4. Metformin and Lafora Disease

Lafora disease (OMIM 254780) is an ultra-rare form of autosomal, recessive progressive myoclonic epilepsy (PME), characterized by the accumulation of insoluble glycogen-like inclusions (polyglucosans) in the brain and other tissues; these are called Lafora bodies (LBs), and they can be identified with periodic acid Schiff (PAS) staining [80,81,82,83,84,85,86]. Lafora is a neurodegenerative disease, also considered a glycogen storage disease [87,88,89,90,91,92]. Various genetically engineered animal models, showing multiple symptoms present in patients with the disease, have been used to study the molecular basis of the disorder and search for effective therapies [93,94,95,96,97]. One of the compounds that successfully improved their symptoms is metformin [26,98], and as we will see below, it is already being used in clinical practice.

### 4.1. Clinical Aspects of Lafora Disease

Lafora disease is a fatal neurological disorder that usually begins in children between the ages of 10 and 15 who appear to have normal neurological development. The first symptoms are epileptic seizures, myoclonus, and/or cognitive alterations that cause school difficulties [86,99]. The cognitive and neurological deterioration is very rapid, and very soon language and intellectual problems occur that continue to worsen until patients develop severe dementia, ataxia, dysarthria, amaurosis, and respiratory failure. There are no specific treatments for the disease; seizures alone are treated with anticonvulsant drugs, but patients quickly develop resistance and myoclonus becomes constant. Status epilepticus or aspiration pneumonitis usually leads to death within the next 5 to 10 years from diagnosis [86,100,101].

Mutations in *EPM2A* (epilepsy of progressive myoclonus type 2 gene A) or *NHLRC1*/*EPM2B* (NHL repeat-containing protein 1/epilepsy of progressive myoclonus type 2 gene B) [102,103,104,105], encoding the laforin or malin proteins, respectively [106,107,108], have been described as causes of the disease. Lafora disease-causing mutations in the *EPM2A* gene represent approximately 60% and in the *EPM2B* gene 35% of patients with Lafora disease. Patients with mutations in the *EPM2B* gene appear to have a slightly milder phenotype than patients with mutations in the *EPM2A* gene [109]. In addition, mutations in the same gene and even the same mutation have been described to be associated with important phenotypic variations. Therefore, it has been suggested that genetic or epigenetic modifying factors could be responsible for the age of onset and severity of the disease [110].

Laforin and malin form a functional complex and work together in the regulation of glycogen synthesis, in the homeostasis of glucose transporters, in the maintenance of proteostasis, and in the response to oxidative stress, among other physiological pathways (reviewed in [111]).

### 4.2. Animal Models of Lafora Disease

Several experimental models of Lafora disease have been generated by deleting the *Epm2a* or *Epm2b* genes in mice. We and other groups have analyzed the functional alterations of two of them: *Epm2a^−/−^* [93] and *Epm2b^−/−^* [97] mice. Both models present memory deficiencies, impaired motor activity and coordination, dyskinesia, altered neuronal excitability, and myoclonus similar to those present in patients with the disease [93,97,112,113]. The presence of neurodegenerative processes and the accumulation of Lafora bodies in the brain correlate with the appearance of functional abnormalities and with the presence of reactive astrogliosis, oxidative stress, altered proteostasis, and impaired autophagy [30,97,114,115,116,117,118,119,120,121].

### 4.3. Pharmacological Interventions in Animal Models of Lafora Disease

We have studied the effect of various pharmacological treatments in the *Epm2b^−/−^* mouse model [26,98,122]. To improve proteostasis, we use 4-phenylbutyric acid (4-PBA), a chemical chaperone that reverses the misfolding and aggregation of proteins associated with various neurodegenerative diseases [123,124]; trehalose, another chemical chaperone that prevents protein denaturation and protects cellular integrity against stress phenomena [125]; and sodium selenate, an agent that reduces oxidative stress and the appearance of epileptic seizures in other animal models [126,127]. Our results indicate that, of the substances used, 4-PBA and sodium selenate, considerably improve memory impairment, motor activity, abnormal posture, and dyskinesia, as well as sensitivity to PTZ [26,98,122].

We also use metformin in *Epm2b^−/−^* mice, and showed that it reduces the accumulation of polyglucosans and polyubiquitin aggregates in the brain, decreases neuronal loss and reactive astrogliosis, and improves neuropsychological tests [26]. In addition, metformin decreases susceptibility to seizures, reduces the number and length of seizures, and eliminates the mortality induced by the pro-convulsive agent pentylenetetrazol (PTZ) in *Epm2a^−/−^* and *Epm2b^−/−^* mice [98]. These results allowed the designation of metformin as an orphan drug for the treatment of Lafora disease by the European Medicines Agency (EMA) in 2016 and the United States Food and Drug Administration (FDA) in 2017.

Due to these regulatory authorizations, metformin has been introduced into the clinical treatment of patients with Lafora disease. In a study with 10 patients, the authors indicated that the result of the metformin administration was inconclusive, probably because the patients engaged in the trial were quite advanced in the disease. In any case, the authors suggested that treatments should be attempted as early as possible in the course of Lafora disease [128].

## 5. Conclusions

**Figure 2:**
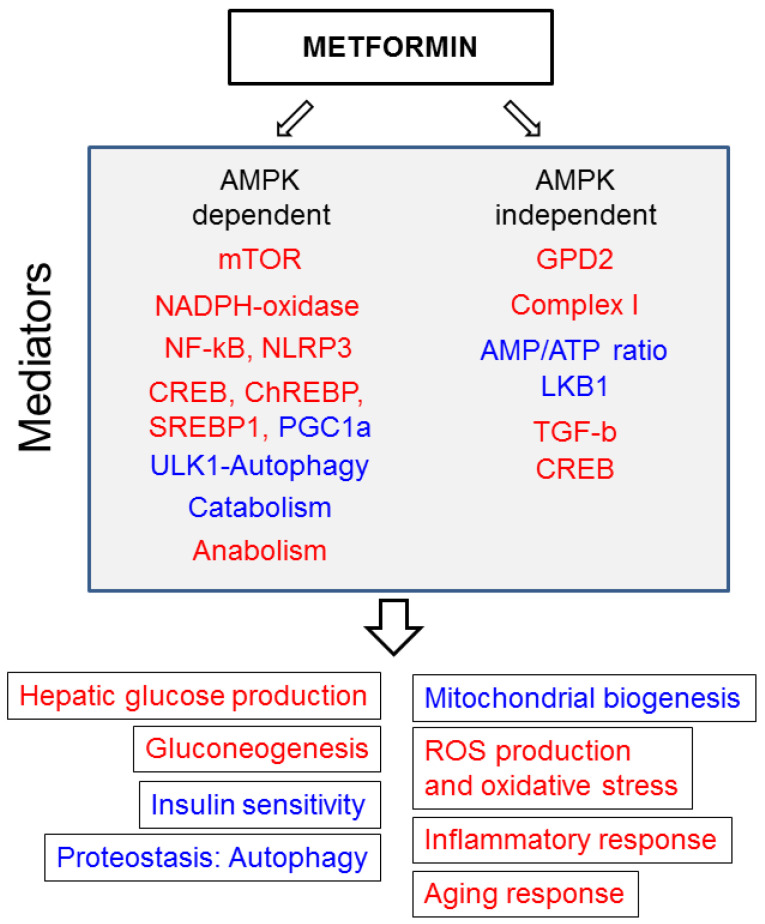
Mechanisms of action of metformin and cellular outcomes. Proteins affected by metformin, either via AMPK-dependent and AMPK-independent pathways, are indicated. In red are outcomes that are inhibited by metformin. In blue are those that are enhanced by metformin.

In conclusion, metformin has beneficial effects on several neurological disorders that could be attributed to both AMPK-dependent and AMPK-independent mechanisms of action (Figure 2).

As a whole, metformin improves mitochondrial function (thereby reducing ROS production and oxidative stress), reduces the inflammatory response, reduces glucose production, and improves proteostasis (enhances the degradation of toxic aggregates). Therefore, the use of metformin as a disease-modifying drug is widely recognized. However, as the regular dose of metformin is 1–2 g/day, future studies should be aimed to understand what is the main mechanism of action related to neuroprotection, in order to look for more active compounds that affect only this pathway.

## Data Availability

Not applicable.
